# Low calpain-9 is associated with adverse disease-specific survival following endocrine therapy in breast cancer

**DOI:** 10.1186/1471-2407-14-995

**Published:** 2014-12-23

**Authors:** Jillian Davis, Stewart G Martin, Poulam M Patel, Andrew R Green, Emad A Rakha, Ian O Ellis, Sarah J Storr

**Affiliations:** Academic Clinical Oncology, University of Nottingham, Division of Cancer and Stem Cells, Nottingham University Hospitals NHS Trust, City Hospital Campus, Nottingham, NG5 1 PB UK; Histopathology, University of Nottingham, Division of Cancer and Stem Cells, Nottingham University Hospitals NHS Trust, City Hospital Campus, Nottingham, NG5 1 PB UK

**Keywords:** Calpain, Calpain-9, Breast cancer, Endocrine therapy

## Abstract

**Background:**

The calpains are intracellular cysteine proteases that function in a variety of important cellular functions, including signalling, motility, apoptosis and survival. In breast cancer high calpain-1 and calpain-2 expression has been associated with adverse clinical outcome. Calpain-9 was thought to be exclusively expressed in the digestive tract; however recent studies have shown that this protein is also expressed in breast tissue.

**Methods:**

We investigated the expression of calpain-9 in a large cohort of early stage breast cancer patients (n = 783) using immunohistochemistry on a tissue microarray. Patients had long-term follow-up information available for analysis.

**Results:**

Low expression of calpain-9 was associated with patients over 40 years of age (*P* = 0.025), smaller tumour size (*P* = 0.001), lower tumour stage (*P* = 0.009), a more favourable Nottingham Prognostic Index value (*P* = 0.002) and positive oestrogen receptor status (*P* = 0.014). Calpain-9 expression was not associated with survival in the total patient cohort, however low calpain-9 expression was associated with adverse survival in patients who received endocrine therapy (*P* = 0.033), which remained significant in multivariate Cox regression analysis accounting for potential confounding factors (hazard ratio (HR) = 0.56, 95% confidence interval (95% CI) = 0.36-0.89, *P* = 0.013). Low calpain-9 expression was also associated with adverse survival in patients with an intermediate Nottingham Prognostic Index value (*P* = 0.009), and remained so in multivariate analysis (HR = 0.54, 95% CI = 0.36-0.82, *P* = 0.003).

**Conclusions:**

This study suggests that calpain-9 may play a role in breast cancer and that low expression is associated with poorer patient clinical outcome following endocrine therapy. Validation studies are warranted as determining expression of calpain-9 may provide important prognostic information.

## Background

Breast cancer is a heterogeneous disease; one major factor of disease heterogeneity is tumour expression of the oestrogen receptor (ER). ER positive breast cancers can be treated using endocrine therapies including aromatase inhibitors that inhibit oestrogen synthesis, such as exemestane, letrozole and anastrozole, in post-menopausal women and selective ER modulators that compete with oestrogen for receptor binding, such as tamoxifen, in both post-menopausal and pre-menopausal women. Tumours can acquire resistance to these therapies which is a major obstacle for the successful management of ER positive tumours.

The calpain family is a group of calcium activated intracellular cysteine proteases that function in a number of important cellular processes including, cytoskeletal remodelling, cell signalling and both survival and apoptosis; despite clear roles in a number of important cellular processes, many of the precise physiological functions of calpain, and mechanisms to control proteolytic activity of the enzyme remain to be fully elucidated (reviewed in [1]). Dysregulation of calpain expression has been implicated in numerous disease states such as limb-girdle muscular dystrophy type 2A, Alzheimer’s, ischemia and cancer. The archetypal members of the calpain family are calpain-1 (encoded by *CAPN1*) and calpain-2 (encoded by *CAPN2*), and are the most widely studied due to their ubiquitous expression. Calpain-1 and calpain-2 are composed of different 80 kDa catalytic subunits and require a common 28 kDa regulatory subunit, calpain-4 (encoded by *CAPNS1*), to form a heterodimer. Both calpain-1 and calpain-2 can be inhibited by the endogenous inhibitor calpastatin. Aberrant expression of calpain family members has been implicated in tumour progression in a number of cancers and expression of calpain-1 and calpain-2 in breast cancer has been shown to be important in patient prognosis [2, 3]. In addition to breast cancer, calpain-1 and calpain-2 expression has been shown to be altered in a number of other tumour types such as ovarian, pancreatic and gastric [4–6] (reviewed in [7]). The expression of the archetypical calpains has been implicated in ER signalling, as 17β-oestradiol has been shown to result in activation of calpain [8], and increased activity has been shown in ER positive tumours [9].

Calpain-9 (encoded by *CAPN9*) (also known as nCL-4) is a more recently charaterised member of the calpain family that was originally thought to be expressed in a digestive tract tissue dependent manner. Current evidence suggests that calpain-9 also associates with the common 28 kDa regulatory subunit calpain-4 [10], although co-localisation of calpain-9 and calpain-8 (encoded by *CAPN8*) (also known as nCL-2) is also implicated in the gastrointestinal tract [11]. The crystal structure of mini-calpain-9 compared to that of mini-calpain-1 suggests that the proteases may act on different substrates and have different mechanisms of activation. There are few studies on calpain-9 and its activity and expression in breast cancer; however, in the breast cancer cell line MCF-7, calpain-9 appears to have a role in lumen formation [12]. Gene expression of calpain-9 has been shown to be lowered in gastric cancer; however tissue based protein expression showed no association with survival or pathological variables [4, 13]. In murine NIH3T3 fibroblasts knockdown of calpain-9 expression results in cellular transformation, and tumourigenesis [14]. This study aimed to investigate the expression of calpain-9 in breast cancer and to determine links with pathological variables of tumours and patient prognosis; in particular we aimed to investigate the importance of calpain-9 in patient subgroups including those that received endocrine therapy.

## Methods

### Study patients

This study is reported in accordance with REMARK criteria [15] and was approved by Nottingham Research Ethics committee 2 under the title ‘Development of a molecular genetic classification of breast cancer’ (REC202313). This retrospective study utilised 4 μm sections of a tissue microarray (TMA) consisting of 0.6 mm cores from 783 patients on a poly-L-lysine slides. The cohort was comprised of a well characterised group of early stage invasive breast cancer patients that were treated at Nottingham University Hospitals, between 1987 and 1998 and had long term follow-up information available. The median age of the cohort was 55 years (ranging from 18–72), 59.0% (462/783) of patients had stage I disease, 49.6% (388/783) of patients had grade 3 tumours, and 50.4% (395/783) of patients had an intermediate Nottingham Prognostic Index (NPI) value. Information on clinical history and outcome is prospectively maintained and the patients were assessed in a standardised manner for clinical history and tumour characteristics. Data was available on a wide range of biomarkers; ER, progesterone receptor (PgR), HER2 status and lymphovascular invasion (LVI) determined by immunohistochemistry were available for this cohort and have been described previously [16]. Tumours were classified as basal phenotype if cytokeratin (CK)-5/6 and/or CK-14 expression was above 10%. Breast cancer-specific survival was defined as the time interval between primary surgery to death from breast cancer in months. Similarly relapse-free survival was defined as the time interval from primary treatment to date of disease relapse in months. Patients were managed in a standard manner, where all patients underwent a mastectomy or wide local excision, as decided by disease characteristics or patient choice, followed by radiotherapy if indicated. Patients received systemic adjuvant treatment on the basis of Nottingham Prognostic index (NPI), ER, and menopausal status. Patients with an NPI score less than 3.4 did not receive adjuvant treatment and patients with an NPI score of 3.4 or higher were candidates for CMF chemotherapy (cyclophosphamide, methotrexate and 5-fluorouracil) if they were ER negative or premenopausal; and endocrine therapy if they were ER positive.

### Immunohistochemistry

Immunohistochemistry was performed on slides that were deparaffinised in xylene, followed by rehydration in ethanol. Antigen retrieval was performed in 0.01 mol L^-1^ sodium citrate buffer (pH 6) in a microwave: 750 W for 10 minutes, followed by 450 W for 10 minutes. Staining was achieved using a Novolink Polymer detection kit (Leica Microsystems, Milton Keynes, UK) according to the manufacturers’ instructions. Briefly, Peroxidase block was added to the tissues, which were then washed with Tris-buffered saline, and a protein block solution was added. Anti-calpain-9 antibody (Abnova, Taipei City, Taiwan; clone 3A6) was diluted 1:50 and incubated on the tissues for 24 hours at 4°C. Antibody specificity was demonstrated using Western blotting (data not shown) and has been used in other calpain-9 studies [12]. Following antibody incubation, the tissues were washed and then subject to incubation with a post primary solution. Tissues were then washed and subject to incubation with Novolink polymer solution. Immunohistochemical reactions were developed using 3, 3’ diaminobenzidine as the chromogenic substrate and sections were counterstained with haematoxylin. Following staining, sections were dehydrated and fixed in xylene prior to mounting with DPX. Breast tumour composite sections comprising of tumours grade 1–3 were included as positive and negative controls.

Staining was examined and scored at 200× magnification using immunohistochemical H scoring after scanning of the slides with a Nanozoomer Digital Pathology Scanner (Hamamatsu Photonics, Hertfordshire, UK). Staining was assessed as none (0), weak (1), medium (2), and strong (3) over the percentage area of each staining intensity. H scores were calculated by multiplying the percentage area by the intensity grade (H score range 0–300). Greater than 30% of cores were scored by a second assessor blinded to first assessment scores. Single measure intraclass correlation coefficients between scores for the TMA was 0.77 showing good concordance between scorers.

### Statistical analyses

Unbiased high and low protein expression cut-points were determined using X-Tile software and were determined prior to statistical analyses [17]. The relationship between high and low protein expression and clinicopathological variables was assessed using Pearson Chi Square test of association (χ^2^). Survival curves were plotted according to the Kaplan-Meier method and significance determined using the log-rank test. Multivariate survival analysis was performed by Cox Proportional Hazards regression model. Spearman rank order correlations were performed to test associations between proteins. All differences were deemed statistically significant at the level of *P* < 0.05. Statistical analysis was performed using SPSS 21.0 software (IBM, Armonk, USA).

## Results

### Staining location and frequency

**Figure 1:**
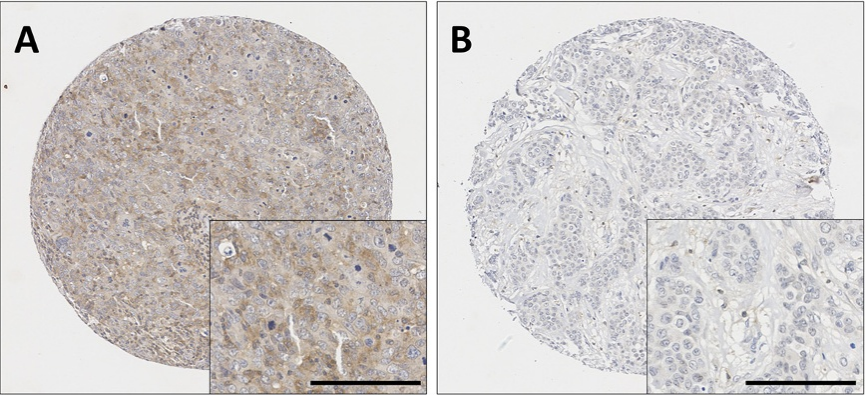
**Representative photomicrographs following immunohistochemical staining of (A) high calpain-9 and (B) low calpain-9 staining.** Photomicrographs are shown at 100x magnification with 200x magnification inset box where scale shows 100 μm.

Calpain-9 demonstrated cytoplasmic staining with heterogeneity in intensity between adjacent tumour cells ranging from weak to strong staining. Calpain-9 staining had a median H-score of 40 and ranged between 0 and 220. Photomicrographs showing representative staining patterns are shown in Figure 1. A total of 783 breast cancer specimens were analysed using a tissue microarray; of which 58.1% (455/783) were invasive ductal carcinomas, 17.0% (133/783) were tubular mixed, 6.5% (51/783) were classic lobular, all other subcategories accounted for less than 5% of the studied cohort. Calpain-9 H-scores were dichotomised using X-Tile software into low and high immunoreactivity based on patient survival; X-tile generated an H score cut point of 40 with 465 (59.4%) cases having a low score. A proportion of sample cores within the TMA were unable to be assessed as they were missing or cores had an insufficient number of tumour cells.

### Relationship with clinicopathological variables

The expression of calpain-9 was tested for its association with a number of clinicopathological variables (Table 1). Low expression of calpain-9 was significantly associated with a number of clinicopathological factors, including; patients over 40 years (χ^2^ = 5.04, d.f. = 1, *P* = 0.025), smaller tumour size (χ^2^ = 10.67, d.f. = 1, *P* = 0.001), smaller tumour stage (χ^2^ = 9.34, d.f. = 2, *P* = 0.009), more favourable Nottingham prognostic index (NPI) values (χ^2^ = 12.96, d.f. = 2, *P* = 0.002), and ER positive tumours (χ^2^ = 6.09, d.f. = 1, *P* = 0.014). No association was observed between the expression of calpain-9 and tumour grade, basal-like phenotype tumours, PgR status, HER2 status or LVI determined by immunohistochemistry.

**Table 1:** **The** ***P*** **values are resultant from Pearson χ** ^**2**^ **test of association or Fishers Exact when an asterisk is included**

Variable	Calpain-9 expression in total patient cohort			Calpain-9 expression in patients that received endocrine therapy		
	Low	High	P value	Low	High	P value
Age						
≤40 years	36 (4.6%)	40 (5.1%)	**0.025**	4 (1.4%)	2 (0.7%)	0.695*
>40 years	429 (54.8%)	278 (35.5%)		160 (54.1%)	130 (43.9%)	
Size						
≤2 cm	296 (38.0%)	166 (21.3%)	**0.001**	88 (29.7%)	56 (18.9%)	0.055
>2 cm	166 (21.3%)	151 (19.4%)		76 (25.7%)	76 (25.7%)	
Stage						
1	294 (37.7%)	168 (21.6%)	**0.009**	70 (23.6%)	53 (17.9%)	
2	128 (16.4%)	115 (14.8%)		73 (24.7%)	64 (21.6%)	0.784
3	39 (5.0%)	35 (4.5%)		21 (7.1%)	15 (5.1%)	
Grade						
1	87 (11.2%)	48 (6.2%)	0.255	8 (2.7%)	8 (2.7%)	0.498
2	155 (19.9%)	101 (13.0%)		53 (17.9%)	50 (16.9%)	
3	220 (28.2%)	168 (21.6%)		103 (34.8%)	74 (25.0%)	
Nottingham prognostic index						
<3.4	158 (20.3%)	71 (9.1%)	**0.002**	7 (2.4%)	7 (2.4%)	0.909
3.4-5.4	219 (28.2)	176 (22.7%)		117 (39.5%)	94 (31.8%)	
>5.4	83 (10.7%)	70 (9.0%)		40 (13.5%)	31 (10.5%)	
Basal status						
Non-basal	351 (46.9%)	240 (46.9%)	0.582	138 (48.6%)	104 (36.6%)	0.257
Basal	90 (12.0%)	68 (9.1%)		20 (7.0%)	22 (7.7%)	
ER status						
Negative	105 (13.8%)	97 (12.8%)	**0.014**	26 (9.0%)	29 (10.0%)	0.180
Positive	345 (45.5%)	212 (27.9%)		134 (46.4%)	100 (34.6%)	
PgR status						
Negative	183 (24.3%)	138 (18.3%)	0.246	64 (22.6%)	51 (18.0%)	0.257
Positive	265 (35.1%)	168 (22.3%)		96 (33.9%)	72 (25.4%)	
HER2 status						
Negative	394 (51.5%)	264 (34.5%)	0.099	131 (45.5%)	111 (38.5%)	0.765
Positive	55 (7.2%)	52 (6.8%)		26 (9.0%)	20 (6.9%)	
Lymphovascular invasion						
Negative	255 (36.9%)	168 (26.1%)	0.490	82 (32.2%)	76 (29.8%)	0.164
Positive	127 (19.7%)	94 (14.6%)		59 (23.1%)	38 (14.9%)	

Significant *P* values are indicated by bold. The immunohistochemistry cohort was comprised of 783 patients; however, scores were not available for every patient for each marker. The number of observations for each marker is shown for each clinicopathological variables and percentage in parentheses. NPI is Nottingham prognostic index, ER is oestrogen receptor, PgR is progesterone receptor.

We further investigated the relationship between the expression of calpain-9 and the expression of other members of the calpain family that had previously been determined using immunohistochemistry in another study [2]. Calpain-9 expression was not significantly associated with calpain-1 expression (r^2^ = 0.04, *P* = 0.283); but was significantly associated with calpastatin expression (r^2^ = 0.13, *P* < 0.001) and calpain-2 expression (r^2^ = -0.12, *P* = 0.001), although with marginal biological relevance.

### Relationship with clinical outcome

**Figure 2:**
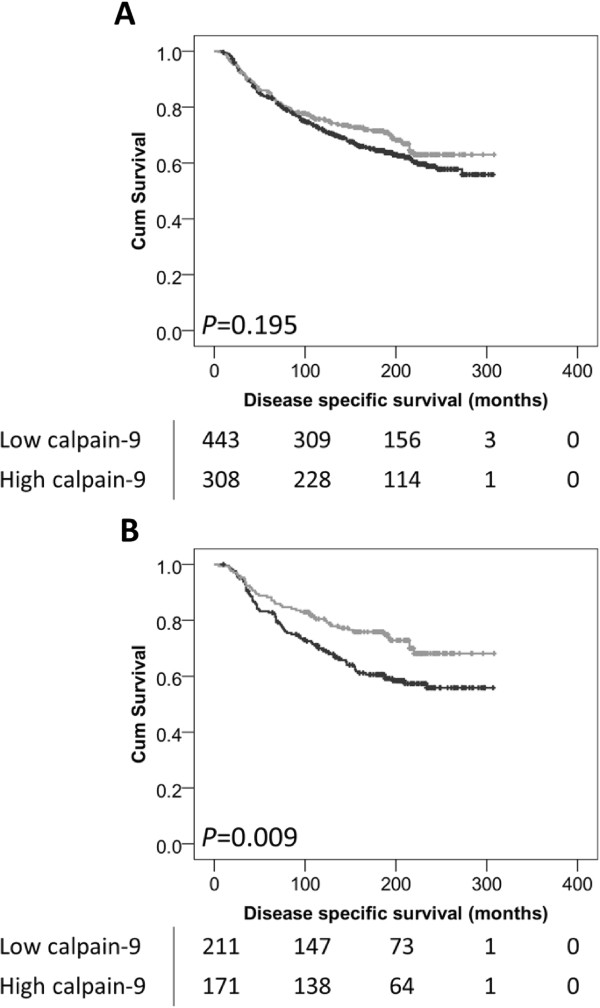
**Kaplan-Meier analysis of breast cancer specific survival showing the impact of low (black line) and high (grey line) calpain-9 expression in the total patient cohort (A), and in those patients with an intermediate NPI value (B); with significance determined using the log-rank test.** The numbers shown below the Kaplan-Meier survival curves are the number of patients at risk at the specified month.

**Figure 3:**
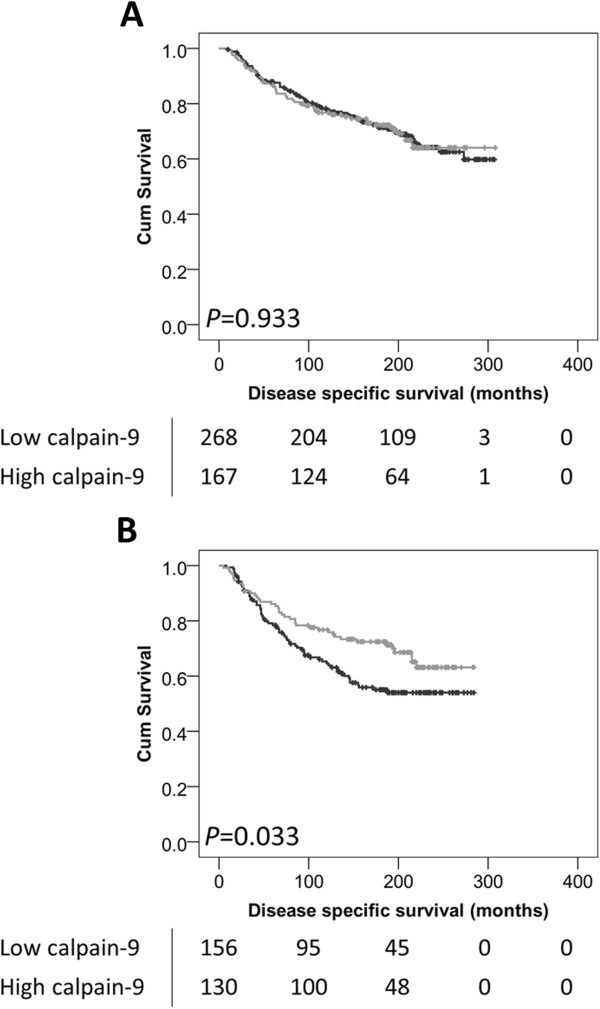
**Kaplan-Meier analysis of breast cancer specific survival showing the impact of low (black line) and high (grey line) calpain-9 expression in patients that did not receive endocrine therapy (A), and in those that did receive endocrine therapy (B); with significance determined using the log-rank test.** The numbers shown below the Kaplan-Meier survival curves are the number of patients at risk at the specified month.

The expression of calpain-9 in invasive breast cancers was not associated with overall disease-specific survival in the total cohort of patients (*P* = 0.195) (Figure 2A). However, low calpain-9 expression was significantly associated with adverse survival in patients with an intermediate NPI value (*P* = 0.009) (Figure 2B), but not in those patients with a good NPI values (*P* = 0.918) or poor NPI values (*P* = 0.464) (data not shown). Calpain-9 expression remained significant for overall disease-specific survival in those patients with an intermediate NPI score (hazard ratio (HR) = 0.54, 95% confidence interval (95% CI) = 0.36-0.82, *P* = 0.003). In the multivariate Cox regression, the potential confounding factors of PgR status and LVI (with individual Kaplan Meier statistics of *P* = 0.027 and *P* < 0.001 respectively) were included in the analysis (Table 2). Perhaps most interestingly, low expression of calpain-9 was associated with adverse overall disease-specific survival in those patients that received endocrine therapy (*P* = 0.033) (Figure 3B). In multivariate Cox regression the expression of calpain-9 remained significant for overall disease-specific survival in those patients that received endocrine therapy (HR = 0.56, 95% CI = 0.36-0.89, *P* = 0.013). In the multivariate Cox regression, the potential confounding factors of tumour size, tumour stage and grade, NPI status, PgR status, HER2 status and LVI determined by immunohistochemistry (with individual Kaplan-Meier statistics of *P* < 0.001 for all variables except tumour size (*P* = 0.036) and HER2 status (*P* = 0.003)) were included in the analysis (Table 2). In addition to these prognostic variables, calpain-1, calpain-2 and calpastatin expression were also included in multivariate Cox regression, where expression of calpain-9 remained significant for disease specific survival (HR = 0.48, 95% CI = 0.30-0.77, *P* = 0.002). Calpain-9 expression was not associated with disease-specific survival of ER positive patients or patients with basal-like disease.

**Table 2:** **Multivariate Cox regression analysis showing calpain-9 expression, various pathological variables and their effect upon disease specific survival in patients treated with endocrine therapy (A) and with an intermediate NPI value (B)**

**A**				
**Multivariate cox regression analysis**				
	**Sig.**	**Exp(B)**	**95.0% CI for Exp(B)**	
			**Lower**	**Upper**
Calpain-9 expression	**0.013**	0.563	0.358	0.885
Tumour size	0.093	1.556	0.929	2.606
Tumour stage	**0.005**	2.135	1.258	3.624
Tumour grade	0.081	1.683	0.937	3.021
NPI	0.854	0.927	0.413	2.081
PgR status	0.204	0.747	0.476	1.172
HER2 status	**0.005**	2.172	1.270	3.716
LVI status	0.130	1.396	0.906	2.151
**B**				
**Multivariate cox regression analysis**				
	**Sig.**	**Exp(B)**	**95.0% CI for Exp(B)**	
			**Lower**	**Upper**
Calpain-9 expression	**0.003**	0.543	0.360	0.817
PgR status	0.076	0.705	0.480	1.037
LVI status	**0.000**	2.474	1.682	3.638

Sig is the *P* value, Exp(B) is the hazard ratio and 95% CI for Exp(B) is the 95% confidence interval. Significant *P* values are indicated in bold.

### Relationship with endocrine therapy

Low calpain-9 expression was associated with traditionally good prognostic markers, however survival of ER positive breast cancer patients treated with endocrine therapy was associated with low calpain-9 expression. We therefore assessed the expression of calpain-9 in breast cancer patients that received endocrine therapy against their clinicopathological data (Table 1). No associations were observed between calpain-9 expression and clinicopathological variables.

## Discussion

This study investigated the expression of the calpain family member calpain-9 in a large consecutive series of early invasive breast cancer specimens using immunohistochemistry. A total of 37% of patients received endocrine therapy, including tamoxifen, or a combination of tamoxifen and goserelin acetate. The expression of calpain-9 in breast cancer has been described *in-vitro* [12]; however, this study clearly demonstrates that calpain-9 is expressed in invasive breast cancer and is not expressed solely in a digestive tract specific manner. Low expression of calpain-9 was associated with patients over 40 years, smaller tumour size and stage, favourable NPI values, and ER positive tumours.

The calpain system in general has been implicated in tumour progression, including altering cellular migration, survival and apoptosis; and expression of calpain-1, calpain-2 and calpastatin have been shown to be important in breast cancer [2, 3, 18]. High calpain-2 expression in breast cancer is associated with poor survival in patients with triple negative or basal-like phenotype tumours; and high expression of calpain-1 can predict response following adjuvant trastuzumab therapy [2, 3]. In addition to breast cancer, expression of the calpain family has been described in a number of solid tumour types [4–6]. Whilst the current study measured the expression of calpain-9 it cannot predict the activity of the enzyme; therefore no conclusions about the effect of calpain-9 activity can be made as a result of this research.

Expression of calpain-9 was significantly associated with overall disease-specific survival in those patients with an intermediate NPI value, whereas it was not associated with survival in those patients with good or poor NPI values. Calpain-9 expression remained significant for overall survival in patients with an intermediate NPI value even when potential confounding factors were included in the analysis. The NPI functions to stratify patients’ risk of 5 year recurrence and is used in decision making regarding chemotherapy. It is calculated from the size of the index lesion, the number of positive lymph nodes and tumour grade. Patients with a high NPI are offered chemotherapy, but it is often difficult to determine the best course of action for those with an intermediate NPI. Often, in these cases decisions are based on the presence of other high risk features, such as patient age, tumour grade, nodal involvement and vascular invasion. Our results show patients with an intermediate NPI had a significantly worse disease-specific survival if their tumours had low expression of calpain-9, which could be potentially examined in these patients to aid decision making on systemic treatment. Furthermore, low expression of calpain-9 was associated with adverse disease-specific survival in those patients that received endocrine therapy. Expression of calpain-9 remained significant for disease-specific survival in this sub group of patients even when potential confounding factors were included in the analysis. There was no association between expression of calpain-9 and disease-specific survival in ER positive patients or patients with basal-like disease. Endocrine therapy is often offered to patients with ER positive disease perceived to have low risk disease on traditional clinicopathological features such as tumour stage and grade, NPI and nodal status. Most tumours that initially respond to endocrine therapies can acquire resistance, which is a major obstacle for the successful management of ER positive tumours. Interestingly, although tumours become resistant to endocrine therapy, they can still retain ER expression (reviewed in [19]). The expression of calpain family members has been implicated in ER signalling. An increase in calpain activity has been shown following treatment with 17β-oestradiol, but also in ER positive tumours [8, 9]. There are limited direct reports of ER modulating calpain-9 expression or activity. In lacrimal glands from mice which were treated with 17β-oestradiol and/or progesterone to determine differentially expressed mRNAs, *CAPN9* was shown to be down regulated [20]. Calpain-8 has been shown to be stimulated by 17β-oestradiol in the pituitary, with the calpain-8 promoter containing an oestrogen response element [21].

An interesting observation of this study is that low calpain-9 expression is associated with disease-specific survival, whereas high expression of calpain-1 and calpain-2 are associated with poor breast cancer prognosis in other studies [2, 3]. The implications of this observation are unclear. Low *CAPN9* expression has been observed in gastric cancer; however protein expression of calpain-9 within the tissue showed no association with survival or pathological variables [4, 13]. Interestingly, low calpain-9 expression was associated with adverse survival in breast cancer patients treated with endocrine therapy, whereas low expression of calpain-9 was associated with markers of good prognosis in the total patient cohort. To investigate this further we determined the relationship between calpain-9 expression and clinicopathological variables in those patients treated with endocrine therapy and did not see the same associations. Similar findings were observed for calpain-9 expression in those patients with intermediate NPI values (data not shown). It is unclear why low calpain-9 expression is associated with favourable prognostic factors in the total patient cohort, but with adverse disease-specific survival of patients treated with endocrine therapy.

## Conclusions

This study has demonstrated that the expression of calpain-9 is associated with important clinicopathological variables, and that expression is associated with disease-specific survival in sub-groups of patients. The expression of calpain-9 was associated with disease-specific survival in patients with an intermediate NPI value, and in those patients that received endocrine therapy. As part of future research, these results require validation in multi-centre cohorts of breast cancer patients to determine the clinical utility of measuring calpain-9 expression as an indicator of response to endocrine therapy or in those patients with an intermediate NPI value.
